# Identification of neovascularization by contrast–enhanced ultrasound to detect unstable carotid stenosis

**DOI:** 10.1371/journal.pone.0175331

**Published:** 2017-04-07

**Authors:** Charlotte Schmidt, Thomas Fischer, Ralph-Ingo Rückert, Timm Oberwahrenbrock, Lutz Harms, Golo Kronenberg, Hagen Kunte

**Affiliations:** 1 Charité Center 15 for Neurology, Neurosurgery and Psychiatry, Charité –Universitätsmedizin Berlin, Berlin, Germany; 2 Institute of Radiology, Charité –Universitätsmedizin Berlin, Berlin, Germany; 3 Franziskus Krankenhaus Berlin, Berlin, Germany; 4 NeuroCure Clinical Research Center, Charité-Universitätsmedizin Berlin, Berlin, Germany; 5 Klinik und Poliklinik für Psychiatrie und Psychotherapie, Zentrum für Nervenheilkunde, Universitätsmedizin Rostock, Rostock, Germany; 6 MSB Medical School Berlin, Berlin, Germany; Monash University, AUSTRALIA

## Abstract

**Background:**

Plaque neovascularization accompanies local inflammation and critically contributes to plaque instability. Correct identification of intraplaque neovascularization by contrast–enhanced ultrasound (CEUS) may provide an additional risk marker in carotid stenosis. This pilot study investigates the correlation between histological evaluation of carotid plaque specimens and pre-surgery CEUS to identify neovascularization.

**Methods:**

17 patients with high-grade internal carotid artery (ICA) stenosis were studied. CEUS was performed in all patients shortly before carotid endarterectomy. Neovascularization, infiltration of T cells and macrophages along with intraplaque hemorrhage were studied in excised plaques by immunohistochemistry. Ultrasound-based four-level and two-level classification systems for neovascularization were used. CEUS findings were compared with histological findings.

**Results:**

Scores on the CEUS-based four-level and two-level classifications were robustly correlated with the density of intraplaque vessels (r = 0.635, p = 0.006 and r = 0.578, p = 0.015, respectively). Histological evaluation of regions with strong and prolonged intraplaque enhancement typically showed strong intraplaque neovascularization in conjunction with acute intraplaque hemorrhage. Moreover, higher grades of intraplaque neovascularization as determined by ultrasound were associated with a higher percentage of macrophage-rich areas.

**Conclusion:**

CEUS is a technique well suited to gauge the degree of neovascularization of carotid plaques. Future research will have to define the reliability and validity of CEUS in everyday clinical practice. Further, our study suggests that CEUS may also be useful to pick up features of vulnerable plaques such as acute intraplaque hemorrhages.

## Introduction

Carotid stenosis may precipitate life-threatening complications such as ischemic stroke [1]. The rupture or erosion of a carotid plaque may lead to thromboemboli that occlude vessels in a distal branch. Stroke risk in patients with carotid plaques may vary widely [2]. Well-established risk predictors are the degree of internal carotid artery (ICA) stenosis as well as prior ischemic events in the territory of the internal carotid artery [3,4]. In symptomatic ICA stenosis, the risk of a further ischemic event within the subsequent 12 weeks may be as high as 32%. By contrast, the risk of suffering an ischemic stroke as a result of asymptomatic ICA stenosis ranges between 1 and 2% annually [5,6]. Typical characteristics of high-risk stenosis include a thin fibrous cap alongside a large necrotic core, intraplaque hemorrhage as well as high numbers of infiltrated inflammatory cells [7–11]. Inflammation plays a key role in plaque progression and vulnerability to rupture [7–9]. High intraplaque neovascularization seems to be an important mechanism that sustains local inflammation and may thus serve as an indirect marker of plaque inflammation. In this context, it is not surprising that a number of studies have shown that high plaque neovascularization is associated with a higher risk of symptomatic stenosis [9–11].

Nowadays, ultrasound is able to capture and discern many properties of carotid plaques that might have previously gone undetected [12–17]. Importantly, there appears to be a good correlation between ultrasound imaging and subsequent histopathological findings [2,11,18,19]. In particular, ultrasound investigations into intraplaque neovascularization have garnered increasing attention since intraplaque neovascularization holds promise as an indirect marker of the amount of infiltrated inflammatory cells [2,11,19].

Regarding the use of the CEUS technique, several advantages are worth emphasizing: CEUS is a mobile and a relatively low-cost technique that is suitable for almost every patient. Other techniques such as MRI have more contraindications and are more difficult to conduct, e.g. in patients with claustrophobia, a pacemaker, patients who require intensive care etc. Finally, CEUS is a technique which can be learnt relatively easily.

This study aims to test the hypothesis that contrast-enhanced ultrasound (CEUS) of carotid stenosis is able to quantify intraplaque neovascularization. CEUS was performed in patients before carotid endarterectomy (CEA). Plaque specimens obtained during CEA were then examined using immunohistochemistry.

## Methods

### Patients

Nineteen patients with high-grade carotid stenosis who were about to undergo CEA were enrolled into the study. One patient withdrew his consent prior to scheduled CEA. The carotid specimen of another patient was so calcified that, despite multiple decalcification steps, it could not be processed further. In total, 17 patients completed the study. Patients were classified according to the North American Symptomatic Endarterectomy Trial criteria (NASCET) as having asymptomatic or symptomatic ICA stenosis [5]. Symptomatic ICA stenosis is defined by the occurrence of neurologic symptoms referable to the ipsilateral carotid artery within the preceding 120 days. Other potential causes of stroke such as cardiac embolism had to be ruled out. None of the patients suffering from asymptomatic ICA stenosis had suffered a previous ischemic event attributable to carotid stenosis [5,6].

All patients were on antiplatelet drugs and received a statin. No patient received immunosuppressive treatment (e.g., corticosteroids, azathioprine, monoclonal antibodies etc.). Approval for this study was obtained from the institutional ethics committee of Charité Hospital Berlin (Ethikkommission 1). Informed consent was obtained from all participants prior to enrollment.

### Contrast-enhanced ultrasound imaging of carotid stenosis

CEUS was performed using the AplioTM 500 ultrasound system and a 5-MHz wideband transducer for contrast harmonic imaging (Toshiba, Otawara, Japan) after a bolus injection of 2.4 ml SonoVue echocontrast agent (Bracco, Milan, Italy). Directly after the injection of the contrast agent, the i.v. access was flushed with 5 ml 0.9% sodium chloride. The interval between injection of SonoVue and the beginning of CEUS imaging of the plaque ranged between 10 and 60 seconds depending on arrival time in the carotid artery. The digital recording of the dynamic sequence at the level of the carotid bifurcation was started when the contrast agent reached the carotid bifurcation.

Micro Flow Imaging (Toshiba), which employs summation of microbubble signals, was used to study neovascularization. A video clip in longitudinal and transverse orientation was saved for subsequent analysis. Ten frames per second were acquired over a span of 50 seconds. For the separate imaging of the plaque area in the longitudinal and transverse planes, the procedure was repeated after an interval of five minutes. In order to evaluate intraplaque neovascularization, the radiologist only took into account the flow dynamics of contrast agent within the plaque. Vascularization associated with adventitia was disregarded because these structures are not removed by CEA and thus cannot be examined histologically [20]. T. F. performed all CEUS investigations.

**Fig 1:**
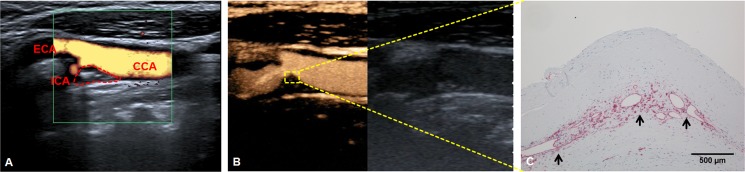
Contrast–enhanced ultrasound (CEUS) for assessing neo-vascularization of carotid plaque. (A) Color Doppler ultrasound of carotid bifurcation. The dotted area represents the stenotic carotid plaque. CCA: Common carotid artery. ICA: Internal carotid artery. ECA: External carotid artery. (B) CEUS of the same area as shown in A. Note yellow-orange color of the contrast agent filling the lumen of the carotid artery. Furthermore, CEUS contrast effects are visible within the carotid plaque (yellow square), indicating plaque neovascularization. (C) Immunohistological evaluation of the plaque area shown in B with anti-CD31 antibody staining. The arrows mark CD31-positive neovessels.

A typical false-color Doppler ultrasound scan of the carotid bifurcation is given in Fig 1. Important anatomical landmarks including the carotid plaque are highlighted. A freeze-frame shot from a CEUS video clip (S1 Video) of the same patient is provided in Fig 1. The flow dynamics of contrast agent within the vessel and plaque are illustrated. Fig 1 then allows the direct comparison of CEUS findings with the histological evaluation (CD31 vessel staining) of the identical area within the carotid plaque of the same patient.

**Fig 2:**
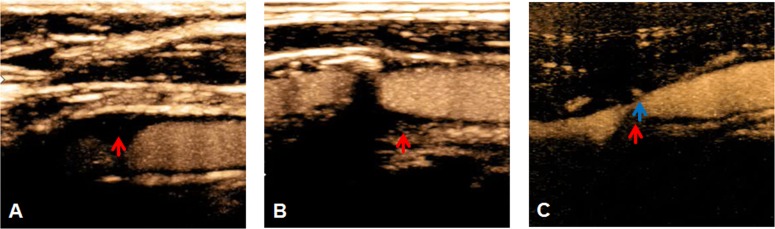
Grades of intraplaque neovascularization as assessed by contrast–enhanced ultrasound (CEUS). The arrows indicate specific plaque characteristics. (A) Grade II: very low diffuse enhancement, no focal intraplaque enhancement. (B) Grade III: clear focal plaque shoulder enhancement in proximal part of carotid plaque. (C) Grade IV: clear focal plaque shoulder enhancement (blue arrow) with strong diffuse intraplaque enhancement in an additional region (red arrow).

Assessment of intraplaque neovascularization followed the classification of Huang and co-workers [21]: Grade I: non-enhancement; grade II: arterial wall vasa vasorum enhancement; grade III: arterial wall vasa vasorum as well as plaque shoulder enhancement; grade IV: extensive and internal plaque enhancement (Fig 2). For clinical simplicity, we dichotomized CEUS findings into carotid stenosis with low (‘A’; grades I and II) and high (‘B’; grades III and IV) intraplaque contrast agent enhancement.

### Tissue preparation, quantification and imaging

**Fig 3:**
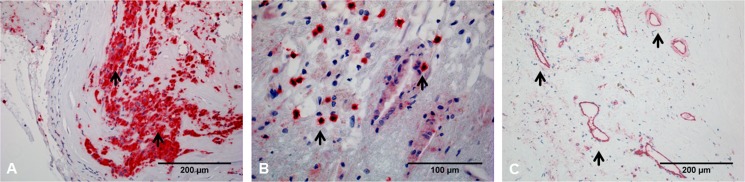
Immunohistochemical evaluation of carotid plaque. Immunohistochemical stainings of macrophages (CD68), T-cells (CD3), and neovascularization (CD31). Arrows highlight distinctive features of the immunohistological staining patterns. (A) Macrophages often appear as confluent infiltrates. (B) T cells typically present as single cells. (C) CD31-positive vessels within atherosclerotic plaque.

CEA specimens were processed as described in detail previously [7]. Briefly, immunohistochemistry of 4 μm paraffin sections followed the alkaline phosphatase method [7]. For immunohistochemical detection of macrophages (CD68), T-cells (CD3), and neovascularization (CD31), specimens were stained automatically using the Ventana Discovery XT staining system (Ventana, Tucson, USA). CD31 is a transmenbrane glycoprotein that is mainly expressed by endothelial cells. Thus, CD31 labels neovascularization within carotid plaques [22]. Additional stains with van Gieson’s and hematoxylin/eosin were performed for verification and spatial classification of immunohistochemical results. For quantitative evaluation, the StereoInvestigator system (Micro Brightfield Europe, Magdeburg, Germany) was used in combination with a spectral confocal microscope and a digital camera (Leica Microsystems, Heidelberg, Germany). As macrophages tend to appear as confluent infiltrates (Fig 3), the total area occupied by macrophages was measured. The density of CD31-positive vessels and the density of T cells (Fig 3) were determined by counting the cells in relation to the total section area (n/mm^2^). The extent of macrophage infiltration was quantified by calculating the percentage of the area occupied by macrophages relative to the total section area.

### Statistical analysis and blinding

Great care was taken to ensure adequate blinding of all investigators. In particular, the histological investigation of CEA specimens (C.S.) and the radiological examination (T.F.) of carotid stenosis occurred entirely independently. Statistical analysis was performed using IBM SPSS Statistics 22 (IBM Corporation, Armonk, U.S.A). Fisher’s exact test was applied to evaluate group differences in gender composition between patients with and patients without symptomatic ICA stenosis. The Mann-Whitney-U test was used to examine differences between groups. Correlations between CEUS grading and histological findings were performed using Spearman’s rho.

## Results

The main clinical characteristics of our sample are summarized in Table 1. The sample consisted of mainly male patients. Median age was 66 years. Seven patients had symptomatic ICA stenosis. The median time interval between CEA and the most recent symptoms related to symptomatic ICA stenosis was 9 days. The median time interval between CEUS and CEA was 1 day. Patients with symptomatic ICA stenosis did not differ from those with asymptomatic stenosis in terms of gender (p = 0.35), age (p = 0.23), the degree of stenosis (p>0.99), or the time interval between the CEUS examination and CEA (p = 0.60).

**Table 1:** Baseline characteristics of the patient sample.

Variables	Patients (n = 17)
Age, years	66 (58–76)
Males,%	10 (58.8)
Grade of ICA stenosis, %	85 (60–95)
Symptomatic ICA stenosis, %	7 (41.2)
Days CEUS to CEA, days	1 (0–9)
Days last symptoms to CEA, days	9 (2–35)

Categorical variables are presented as number (%), continuous variables as median and range (minimum—maximum).

The four-level CEUS classification of intraplaque neovascularization was strongly and positively correlated with increased density of intraplaque vessels on histology (r = 0.635, p = 0.006). Relatively similar results were obtained when the two-level CEUS classification was used (r = 0.578, p = 0.015). Table 2 provides correlations between ultrasound classification of neovascularization and key histological measures.

**Table 2:** Correlations of CEUS-based classification of intraplaque neovascularization and histological parameters.

Variables	US neovasculization grading I—IV	US neovasculization grading A and B
intraplaque vessels, vessels/mm^2^	**0.635 (0.006)**	**0.578 (0.015)**
T cells, cells/mm^2^	0.263 (0.308)	0.452 (0.068)
macrophage rich area, %	**0.499 (0.042)**	**0.503 (0.040)**

Correlations are presented using Spearman’s rho (p).

**Fig 4:**
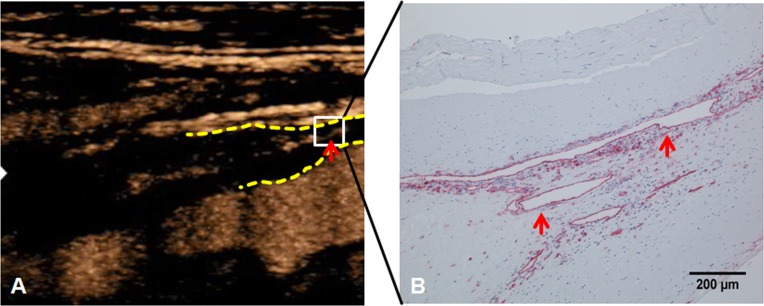
Neovascularization on contrast-enhanced ultrasound (CEUS) and immunohistochemistry. (A) Contrast-enhanced ultrasound (CEUS) of carotid stenosis. The dotted yellow lines mark the proximal beginning of the carotid plaque. The red arrow marks an area of focal neovascularization. (B) The area corresponding to the white square in A was further analyzed with anti-cd31 immunohistochemistry. Note pronounced neovascularization (red arrows) in the surgical specimen.

So far, only few studies by us and others have directly compared CEUS and histopathology [2,18,23]. In this investigation, focal enhancements of contrast corresponded well to intraplaque neovascularization (Figs 1 and 4).

**Fig 5:**
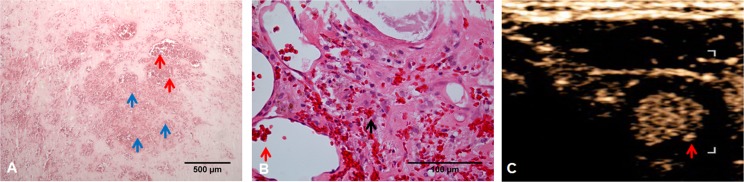
Plaque neovascularization and intra-plaque hemorrhages. (A) Hematoxylin/eosin staining of carotid plaque reveals intraplaque vessels densely filled with erythrocytes (red arrows) alongside acute intraplaque hemorrhages (blue arrows). (B) Higher magnification demonstrates extravascular erythrocytes indicative of intraplaque hemorrhages (black arrow). The intraplaque hemorrhages shown here must be relatively fresh because individual erythrocytes are still clearly demarcated. (C) Axial imaging of carotid artery by contrast-enhanced ultrasound (CEUS). The red arrow points to the area that corresponds to the histological image (A). CEUS is not able to distinguish between contrast agent located within neovessels and contrast agent moving freely within the plaque, such as may be the case in neovessel rupture followed by intraplaque hemorrhage.

Moreover, histological evaluation of regions with intense and prolonged intraplaque enhancement regularly yielded neovascularization and acute intraplaque hemorrhage (Fig 5). CEUS is not able to distinguish between contrast agent located within neovessels and contrast agent moving freely within the plaque, such as may be the case in acute neovessel rupture and subsequent intraplaque hemorrhage.

**Fig 6:**
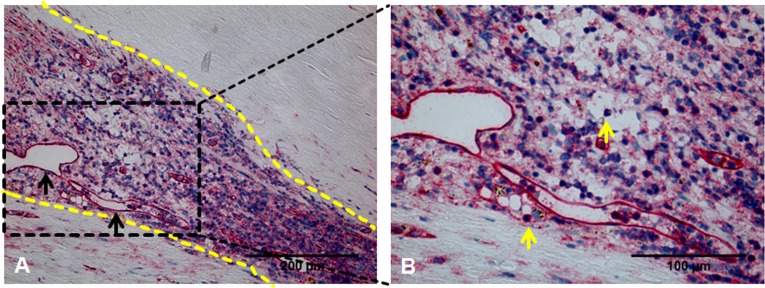
Massive infiltration of inflammatory cells in area of intraplaque neovascularization. (A) CD31 antibody staining reveals intraplaque neovascularization. Black arrows mark individual neovessels. The dotted lines delineate an area within the plaque characterized by a high density of blue-stained nuclei suggesting strong infiltration of inflammatory cells in close proximity to the area of neovascularization. (B) Higher magnification of boxed area shown in A. Yellow arrows mark individual inflammatory cells. Cell shape and nuclear geometry suggest the presence of inflammatory cells (predominantly macrophages and T cells).

An example of massive infiltration of inflammatory cells in the area of neovascularization is given in Fig 6.

Histological results were also compared between patients with symptomatic ICA stenosis and asymptomatic patients (Table 3). The percentage of area infiltrated by macrophages was significantly higher in plaques from patients with symptomatic ICA stenosis. An extended data set containing information on clinical characteristics, immunohistological und CEUS parameters of individual patients is available in S1 Table.

**Table 3:** Comparison of histological and CEUS parameters between asymptomatic patients and patients suffering from symptomatic ICA stenosis.

Variables	Symptomatic (n = 7)	Asymptomatic (n = 10)	p
intraplaque vessels/mm^2^	8.14 (4.33)	6.39 (4.70)	0.109
T cells/mm^2^	41.91 (23.69)	21.91 (16.51)	0.364
macrophage rich area in %	**7.25 (4.31)**	**2.98 (1.00)**	**0.007**
CEUS neovasculization grade I, n(%)	0(0)	0(0)	0.270
CEUS neovasculization grade II, n(%)	1(14.3)	5(50.0)	
CEUS neovasculization grade III, n(%)	5(71.4)	4(40.0)	
CEUS neovasculization grade IV, n(%)	1(14.3)	1(10.0)	
CEUS neovasculization grade A, n(%)	1(14.3)	5(50.0)	0.230
CEUS neovasculization grade B, n(%)	6(85.7)	5(50.0)	

Continuous variables are given as mean (SD). Categorical variables are presented as number (%).

## Discussion

Our pilot study demonstrates that CEUS of carotid stenosis is able to faithfully detect different grades of intraplaque neovascularization confirmed by immunohistochemistry. The percentage of area infiltrated by macrophages was significantly higher in plaques from patients with symptomatic ICA stenosis and in carotid stenosis with higher grades of intraplaque neovascularization as assessed by CEUS. Furthermore, our study suggests that intense and prolonged intraplaque enhancement of ultrasound contrast agent seems to be associated with intraplaque hemorrhage. Intraplaque hemorrhage contributes to plaque instability and plays an essential part in atherosclerotic plaque growth [7,24,25]. Importantly, intraplaque hemorrhage frequently results from the rupture of neovessels which may be caused by, among other factors, high levels of local inflammation. Intraplaque hemorrhage further destabilizes the carotid plaque [26–28]. Increased plaque vascularization seems to be a prerequisite for maintaining the inflammatory process within the plaque. Vasa vasorum are present in the adventitia and, under physiological conditions, nurture the outer components of the vessel wall. The intima, however, is fed by oxygen diffusion from the lumen [9]. If the intima thickens as a result of atherosclerotic progression, oxygen diffusion becomes impaired and the vasa vasorum turn into a major source of nutrients. Hypoxia and inflammation thus stimulate the extent and distribution of the vascular network [29]. Intraplaque neovascularization is based on microvessels originating from the adventitia [26]. These microvessels are immature and fragile and, for this reason, prone to rupture [9]. Furthermore, neovessels are critical for the trafficking of inflammatory cells like T-cells and macrophages into the plaque [9,26]. Intraplaque neovascularization may also be conceptualized as a healing process. In all probability, intraplaque neovascularization is a prerequisite for the removal of amorphous cell material near the lipid core by e.g. macrophages [7,24]. The precise role of plaque neovascularization in the complex pathophysiology of plaque instability and plaque healing remains to be further defined.

CEUS seems to have the potential not only to evaluate intraplaque neovascularization and hemorrhage, but also to examine other features of carotid stenosis. There are studies indicating that microbubbles of ultrasound contrast agent can be conjugated to specific vascular cell adhesion molecules and integrins expressed in endothelial cells [30,31]. Recently, CEUS as a form of molecular imaging has been suggested as a promising technique to evaluate inflammation [2,14]. Microbubbles that are phagocytized by macrophages and can adhere directly to the surface of damaged endothelium may aid in risk stratification of atherosclerotic plaques [32]. Importantly, the grade of neovascularization in CEUS was positively correlated with the size of the macrophage-infiltrated area in our study. Hence, if this result is replicated, CEUS detection of intraplaque neovascularization may also provide the clinician with a measure of macrophage infiltration. Most likely, our observation indicates that high levels of neovascularization promote macrophage infiltration and vice versa.

Algorithms for the automated analysis of the behavior of the ultrasound contrast agent within the plaque tissue may hold great promise. For example, quantification of time-signal intensity curves in CEUS of carotid stenosis may allow the examiner to differentiate between symptomatic and asymptomatic ICA stenosis [24,33]. Still, one has to point out that, in the walls of latex flow phantoms, enhancement patterns typical for vasa vasorum may actually occur in the absence of vasa vasorum [34]. Therefore, algorithms for sorting out artifacts will also have to be developed especially when using automated signal quantification of the contrast agent.

To summarize, our study provides promising initial evidence that contrast-enhanced ultrasound (CEUS) of carotid stenosis may be a useful tool to assess intraplaque neovascularization. However, the technique may require further refinement and testing before it can be broadly implemented in the clinic. Since our pilot study included only a relatively small number of patients, further investigations with a higher number of patients will also be necessary to validate our findings.

## Supporting information

S1 Video Video clip of contrast-enhanced ultrasound (CEUS) investigation. In S1 Video you will find a video clip of the same region like in Fig 1 showing a contrast-enhanced ultrasound (CEUS) investigation of this area. The flow dynamics of contrast agent within the vessel and in the plaque are shown to detect neovascularization.
(MP4)

S1 Table Raw data about patient characteristics, immunohistological and CEUS investigations. (DOCX)
