# Supplementary material for: Identification of neovascularization by contrast–enhanced ultrasound to detect unstable carotid stenosis
Source: PLoS One. 2017 Apr 7;12(4):e0175331. doi: 10.1371/journal.pone.0175331 (PMC5384678; doi:10.1371/journal.pone.0175331)
Supplement: S1 Table — (DOCX) [file pone.0175331.s002.docx]

| ID pts | Area of plaque specimen mm² | T cell count | T/mm² | neo vessel count | neo vessel /mm² | CD68+ area in mm² | CD68+ area/specimen area in % | age | Sympt=1 asympt=0 | stenosis grade | time US until surgeryTage US bis OP | time symptoms until surgery | neo vasc grade US 0-3 = 1-4 |
| --- | --- | --- | --- | --- | --- | --- | --- | --- | --- | --- | --- | --- | --- |
| 1 | 78,742 | 3184 | 40,436 | 325 | 4,127 | 4,850127 | 6,16 | 69 | 1 | 85 | 1 | 10 | 2 |
| 2 | 174,106 | 445 | 2,556 | 1490 | 8,558 | 5,145728 | 2,96 | 73 | 0 | 80 | 2 |  | 2 |
| 3 | 244,293 | 337 | 1,379 | 154 | 0,63 | 9,018028 | 3,691 | 76 | 0 | 60 | 2 |  | 1 |
| 4 | 42,469 | 356 | 8,383 | 256 | 6,028 | 1,88358 | 4,435 | 67 | 0 | 80 | 1 |  | 1 |
| 5 | 30,965 | 657 | 21,218 | 548 | 17,697 | 1,24783 | 4,03 | 76 | 0 | 85 | 1 |  | 3 |
| 6 | 152,608 | 5116 | 33,524 | 1925 | 12,614 | 8,379259 | 5,491 | 66 | 1 | 95 | 0 | 6 | 2 |
| 7 | 106,269 | 3362 | 31,637 | 994 | 9,354 | 1,994838 | 1,877 | 75 | 0 | 90 | 8 |  | 2 |
| 8 | 76,212 | 5472 | 71,8 | 1173 | 15,391 | 8,507497 | 11,163 | 61 | 1 | 80 | 1 | 9 | 2 |
| 9 | 76,016 | 961 | 12,642 | 431 | 5,67 | 0,900316 | 1,184 | 58 | 0 | 80 | 1 |  | 1 |
| 10 | 46,934 | 516 | 10,994 |  | 8,34 | 2,688828 | 5,729 | 64 | 1 | 90 | 9 | 35 | 3 |
| 11 | 78,211 | 3932 | 50,274 | 347 | 4,437 | 2,813634 | 3,597 | 61 | 0 | 95 | 0 |  | 2 |
| 12 | 38,497 | 980 | 25,457 | 149 | 3,87 | 1,007423 | 2,617 | 69 | 0 | 80 | 3 |  | 2 |
| 13 | 101,354 | 4432 | 43,728 | 341 | 3,364 | 2,539083 | 2,505 | 65 | 0 | 85 | 1 |  | 1 |
| 14 | 47,495 | 3392 | 71,418 | 287 | 6,043 | 7,100453 | 14,95 | 75 | 1 | 85 | 4 | 14 | 2 |
| 15 | 44,59 | 972 | 21,799 | 192 | 4,306 | 1,292593 | 2,9 | 65 | 0 | 85 | 2 |  | 1 |
| 16 | 114,022 | 5335 | 46,789 | 724 | 6,35 | 5,696604 | 4,996 | 60 | 1 | 75 | 0 | 2 | 2 |
| 17 | 91,189 | 1681 | 18,434 | 377 | 4,134 | 2,021717 | 2,217 | 58 | 1 | 70 | 1 | 3 | 1 |
